# The Impact of the Intracellular Domains of Chimeric Antigenic Receptors on the Properties of CAR T-cells

**DOI:** 10.32607/actanaturae.27728

**Published:** 2025

**Authors:** D. V. Volkov, V. M. Stepanova, I. A. Yaroshevich, A. G. Gabibov, Y. P. Rubtsov

**Affiliations:** Shemyakin-Ovchinnikov Institute of Bioorganic Chemistry, Russian Academy of Sciences, Moscow, 117997 Russia; Lomonosov Moscow State University, Moscow, 119991 Russia

**Keywords:** CAR T-cell, costimulatory molecules, CD3, intracellular signaling, T-cell receptors

## Abstract

The advent of the T-cell engineering technology using chimeric antigen receptors (CARs) has revolutionized the treatment of hematologic malignancies and reoriented the direction of research in the field of immune cell engineering and immunotherapy. Regrettably, the effectiveness of CAR T-cell therapy in specific instances of hematologic malignancies and solid tumors is limited by a number of factors. These include (1) an excessive or insufficient CAR T-cell response, possibly a result of both resistance within the tumor cells or the microenvironment and the suboptimal structural and functional organization of the chimeric receptor; (2) a less-than-optimal functional phenotype of the final CAR T-cell product, which is a direct consequence of the manufacturing and expansion processes used to produce CAR T-cells; and (3) the lack of an adequate CAR T-cell control system post-administration to the patient. Consequently, current research efforts focus on optimizing the CAR structure, improving production technologies, and further developing CAR T-cell modifications. Optimizing the CAR structure to enhance the function of modified cells is a primary strategy in improving the efficacy of CAR T-cell therapy. Since the emergence of the first CAR T-cells, five generations of CARs have been developed, employing both novel combinations of signaling and structural domains within a single molecule and new systems of multiple chimeric molecules presented simultaneously on the T-cell surface. A well thought-out combination of CAR components should ensure high receptor sensitivity to the antigen, the formation of a stable immune synapse (IS), effective costimulation, and productive CAR T-cell activation. Integrating cutting-edge technologies – specifically machine learning that helps predict the structure and properties of a three-dimensional biopolymer, combined with high-throughput sequencing and omics approaches – offers new possibilities for the targeted modification of the CAR structure. Of crucial importance is the selection of specific modifications and combinations of costimulatory and signaling domains to enhance CAR T-cell cytotoxicity, proliferation, and persistence. This review provides insights into recent advancements in CAR optimization, with particular emphasis on modifications designed to enhance the therapeutic functionality of CAR T-cells.

## INTRODUCTION

**Fig. 1:**
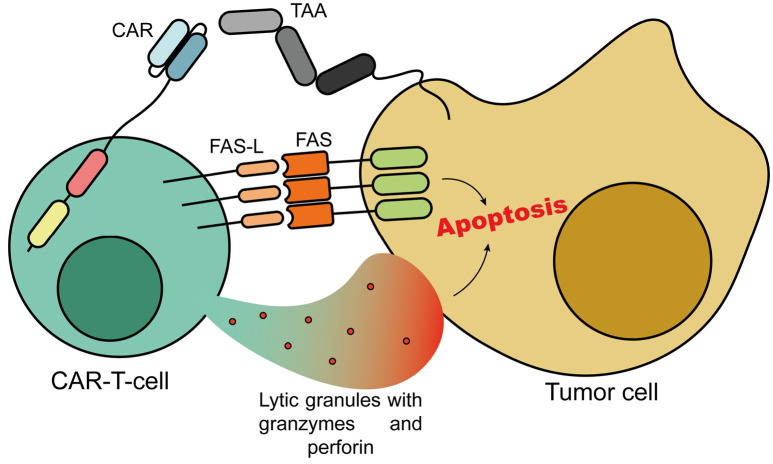
The mechanistic basis of CAR T-cell therapy. The interaction between a CAR T-cell and a tumor cell is enabled by the specific recognition of a tumor-associated antigen (TAA) by the chimeric receptor. This leads to the activation of the cytotoxic functions of the CAR T-cell, mediated by the release of lytic granules containing granzymes and perforin, as well as by the interaction between the Fas ligand (FASL) and Fas receptor (FAS). Consequently, apoptosis of the tumor cell is induced

Today, conventional methods of treating tumors – chemotherapy and radiation therapy – are frequently integrated with comparatively novel immunotherapeutic approaches. These include therapy with monoclonal antibodies and bispecific T-cell engagers, as well as cell therapy, notably with CAR T-cells, which is the focus of this review. The growing interest in more specific, or so-called targeted, therapies has largely to do with the low effectiveness and severe adverse effects of conventional treatments (e.g., systemic genotoxicity) [1], as well as the growing potential shown by novel methods, as particularly well exemplified by the CAR T-cell technology, in treating hematologic diseases [2]. The mechanism of CAR T-cell therapy is based on the recognition of surface markers on tumor cells by cytotoxic CAR T-cells (Fig. 1).

This capability is made possible by the CAR, which comprises three primary domains: an extracellular domain responsible for antigen recognition and the mobility of the recognition moiety, a transmembrane domain involved in immune synapse (IS) formation, and an intracellular domain containing costimulatory and signaling domains that determine the entire spectrum of CAR T-cell responses upon specific activation by antigen binding. Furthermore, the off-tumor toxicity of such immunotherapeutic agents is significantly lower compared to conventional therapies [3]. Additionally, successful CAR T-cell therapy can lead to the formation of a specific memory cell population, ensuring long-term remission [4].

Unfortunately, despite individual successes with CAR T-cells, there remain patients for whom current CAR T-cell therapy provides only temporary relief due to insufficient effect duration or cytotoxicity of the highly personalized cell products. Therefore, research is focused on enhancing the efficacy of CAR T-cell therapy. Some of the key factors in this endeavor are enhancing the efficiency of signal transduction from the membrane-bound CAR into the cell, which activates the transcriptional programs responsible for cytotoxicity, and ensuring the survival of activated cells, their proliferation, the secretion of cytokines and lytic granules, the metabolism, and other functions. This signal transduction is controlled by the intracellular domains (ICDs) of the CAR, and the optimization of their structure is the subject of this review.

## COSTIMULATION DOMAINS

**Fig. 2:**
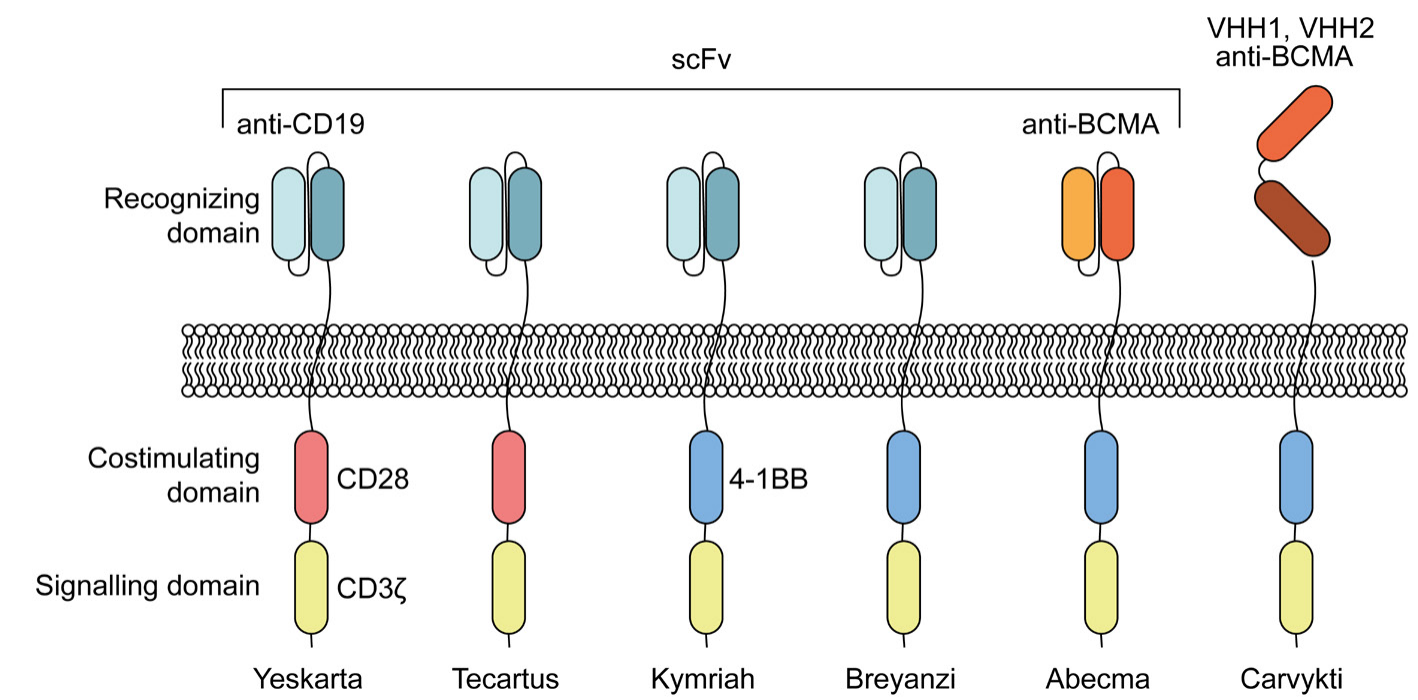
Approved CAR T-cell preparations. Presented are the key domains that mediate recognition and signal transduction to intracellular partners. scFv – single-chain variable fragment; VHH – heavy chain variable domain (nanobody); CD – cluster of differentiation; BCMA – B-cell maturation antigen

In clinically approved CAR T-cell preparations, the function of costimulatory domains is executed by the intracellular components of well-characterized costimulatory T-cell membrane molecules: CD28 and 4-1BB [5] (*Fig. 2*).

**Fig. 3:**
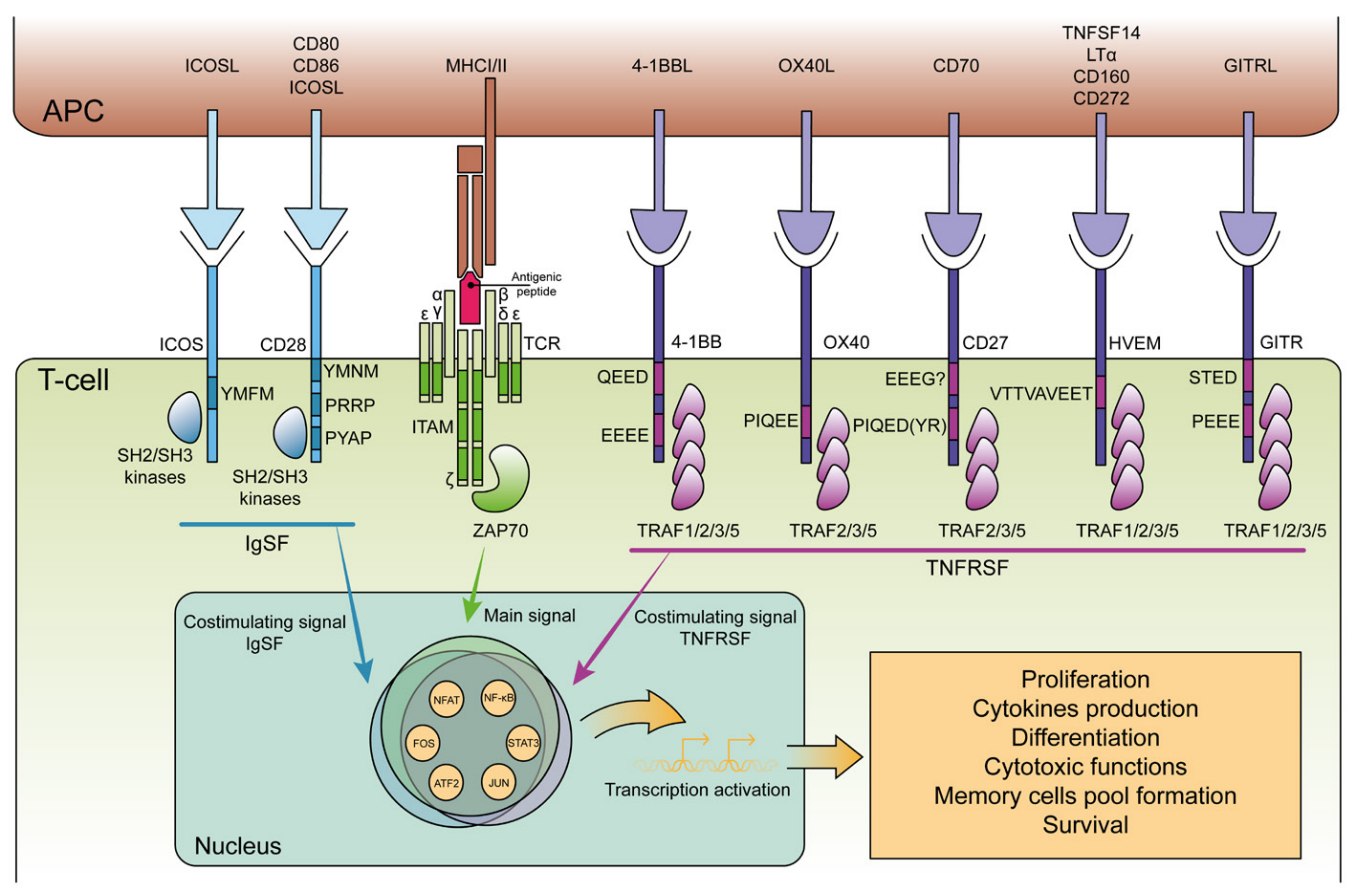
Key superfamilies of T-cell costimulatory receptors. Presented is a general scheme of T-cell activation requiring both the primary and costimulatory signals, with the latter provided by TCR engagement with the major histocompatibility complex and by activating receptors from the IgSF and TNFRSF superfamilies binding their respective ligands. Amino acid sequences that are labeled in the receptors identify the primary signaling motifs. APC – an antigen-presenting cell; MHC I/II – major histocompatibility complex class I/II; IgSF – immunoglobulin superfamily; TNFRSF – tumor necrosis factor receptor superfamily; TNFSF – tumor necrosis factor superfamily; TCR – T-cell receptor; α, β – TCR recognition chains; ζ, γ, δ, ε – CD3 proteins of the TCR complex; ITAM – immunoreceptor tyrosine-based activation motif

These membrane proteins are classified into two superfamilies: the immunoglobulin superfamily (IgSF) and the tumor necrosis factor receptor superfamily (TNFRSF). CARs incorporating other costimulatory domains from these same families, such as ICOS, OX40, CD27, and others, are currently at various stages of development (*Fig. 3*).

**Immunoglobulin superfamily**

Among receptors related to the IgSF, CD28 and ICOS serve as T-cell stimulators. This is attributed to a conserved YXXM motif (where X is any amino acid) which contains a tyrosine (Y) residue that undergoes phosphorylation during activation. This phosphorylation encourages interactions with intracellular signaling partners, including various kinases.

*CD28. *CD28 was the first costimulatory molecule used to generate modified T-cells containing second-generation CARs [6]. These cells demonstrated capability in effect duration and cytokine secretion compared to first-generation cells containing only the CD3ζ signaling domain [7]. CD28 signals are crucial for the activation of naïve T-cells (Tn), since they prevent anergy [8] and promote cytokine secretion, T-cell proliferation, and effector cell differentiation. CD28 is activated by interaction with several ligands, specifically CD80 (B7-1), CD86 (B7-2), and B7-H2 (ICOSL), the latter also an ICOS ligand. Functional motifs within the intracellular part of CD28, proximal (YMNM, PRRP) and distal (PYAP) (*Fig. 3*), bind kinases with SH2 and/or SH3 domains (YMNM – SHIP1, SLP76, GRAP, CBL, PI3K, GRB2, and GADS; PRRP – ITK; PYAP – PDK1, PKCθ, GRB2, STS1/2, CIN85, CD2AP, LCK, and FLNA). The binding of kinases to the costimulator causes conformational changes, their activation, and subsequent interactions with the downstream elements of signaling cascades. As a result, the transcription factors NFAT, AP-1, and NF-κB are activated, and they are associated with interleukin- 2 (IL-2) synthesis and the stimulation of Bcl-XL. Simultaneously, this stimulates T-cell metabolism, increasing aerobic glycolysis, nutrient supply, and anabolic processes [8, 9, 10].

The CAR construct utilizes ICD CD28, which, upon antigen binding to the chimeric receptor, leads to the activation of the PI3K/AKT pathway, enhancing aerobic glycolysis, which positively affects effector T-cells [11]. At the same time, high levels of glycolysis provoke cell exhaustion and reduce cell persistence [12]. To address these challenges, investigations into the consequences of CD28 functional motif mutations are underway. At the same time, mutations in each motif can have an impact on the characteristics of the resulting CAR T-cells. For example, in a pancreatic tumor xenograft model, substitution of the YMNM motif with YMFM in SS1 CAR T-cells targeting mesothelin and based on CD28 modulation decreased the level of CD28 interaction with GRB2. This resulted in reduced signaling through VAV1, diminished calcium current, and attenuated NFAT hyperactivation, thereby decreasing T-cell depletion and dysfunction while increasing T-cell persistence and antitumor efficacy [12]. The substitution of ARRA and YFNM for the PRRP and YMNM motifs in CD28, respectively, augments cellular secretion of interferon-gamma (IFN-γ) and tumor necrosis factor-alpha (TNF-α), reduces the levels of depletion-associated transcription factor Nur77, and enhances CD19 CAR T-cell cytotoxicity, thereby facilitating persistent inhibition of tumor development in mice [13]. Kofler et al. demonstrated that replacing the PYAPP segment in CD28 with AYAAA disrupts the interaction of the PYAP motif with LCK kinase. This disruption reduces IL-2 secretion and suppresses its dependent signaling while also weakening IL-2-dependent proliferation of intratumoral regulatory T-cells (Tregs), thereby enhancing the antitumor activity of such CAR T-cells against solid tumors with a high Treg infiltration level [14]. Furthermore, this CD28 modification enhances CAR T-cell proliferation, metabolism, activation, and cytotoxicity targeting the fibroblast activation protein (FAP). In combination with immune checkpoint inhibitors, these cells demonstrate efficacy in eliminating tumors and exhibit an ability to persist for a lengthy time in humanized xenograft mice and patients with malignant pleural mesothelioma, suggesting a potentially high safety profile [15]. Given that the CD28 ICD is frequently integrated into CARs, alongside the transmembrane domain, it is important to note that this enables CARs to form heterodimers with native CD28 [16], leading to sustained signaling and augmented effector functions in the associated CAR T-cells.

*ICOS. *This receptor is expressed at low levels in Tn cells before T-cell receptor (TCR) activation, with increased expression for several hours following activation [17]. The interaction between ICOS and its ligand, ICOSL, promotes T-cell viability through the stimulation of proliferation and differentiation via pathways analogous to CD28. The effects of these receptors on cytokine synthesis and secretion can vary. While CD28 stimulates IL-2 production, ICOS stimulates IL-10, which is more characteristic of Treg cells [18]. In addition to IL-10, ICOS also promotes the release of IFN-γ, TNF-α, IL-5, IL-13, and IL-17, thereby enhancing the characteristics of effector T-cells and the development of naïve T helper cells (Th) into effector cells of the Th1, Th2, and Th17 subtypes [18, 19]. Analogous to CD28, ICOS induces Bcl-XL expression, leading to increased T-cell viability [20]. The functional tyrosine-containing ICOS YMFM motif (*Fig. 3*) interacts with the PI3K regulatory subunit p50α, which induces a more significant PI3K activation when compared to CD28 [21]. Consequently, the stimulation of ICOS results in the phosphorylation of the AKT, PDK1, ERK1/2, and p38 MAPK kinases, along with the activation of the transcription factors NFAT and NF-κB, which distinguishes ICOS from CD28. The latter also involves JNK kinase in the signaling cascade, and it activates the transcription factor c-Jun [17].

Shen et al. were the first to demonstrate the functional activity of ICOS-based CAR T-cells in a mouse model of glioblastoma [22]. It has been suggested that it is the polarization of CD4^+^ CAR T-cells towards Th1 and Th17 due to the involvement of PI3K/AKT and p38 MAPK (and other mechanisms) that enhances their persistence [23], which increases the antitumor activity of CD8^+^ CAR T-cells as well [23]. Before administering CD4^+^ mesoCAR T-cells (targeting mesothelin) to animals, Wyatt et al. performed low-intensity stimulation of these cells using magnetic beads coated with antibodies against CD3 and ICOS (bead-to-cell ratio = 1 : 10), simultaneously achieving Th17 polarization using a cytokine cocktail. This treatment (compared to CD3 and CD28 stimulation) yielded less differentiated CD4^+^ CAR T-cells and shifted their metabolism towards oxidative phosphorylation, which is characteristic of memory T-cells (Tm). These findings emphasize the advantage of ICOS-directed stimulation of CD4^+^ mesoCAR T-cells. In mesothelioma mice, the combination of CD4^+^ Th17 mesoCAR T-cells and CD8^+^ mesoCAR T-cells [24] demonstrated more effective tumor elimination compared to standard activated CAR T-cells. These data underscore the differential involvement of costimulators contingent on the CD4^+^ or CD8^+^ status of CAR T-cells. Optimal costimulation can be achieved by modifying CD4^+^ and CD8^+^ T-cells with CAR genes with different costimulatory domains, a fact to be taken into account when the most effective CAR T-cell product is needed . Analysis of CAR T-cell costimulation by ICOS revealed that substituting YMFM with FMFM leads to a reduction in CAR T-cell costimulation through ICOS. Consequently, the CAR T-cells exhibit diminished secretion levels of the cytokines in question [25]. To date, modifications to the ICD ICOS that would improve its efficacy have not been documented.

**Tumor necrosis factor receptor superfamily**

This expansive superfamily comprises approximately 30 receptors, classified into three primary groups: (1) tumor necrosis factor receptor-associated factor (TRAF) receptors, (2) death receptors, and (3) molecules with or without a non-functional ICD [26]. To date, only intracellular components from group (1) receptors have been used in the CAR structure, including 4-1BB, OX40, CD27, HVEM, and TNFRSF18. The conserved receptor motifs of this group encompass TRAF-binding (P/A/S/T)X(E/Q)E and PXQQXXD, with X representing any amino acid (AA) [27].

*4-1BB. *4-1BB is frequently integrated into CARs during the process of CAR T-cell preparation. Four of the six approved CAR T-cell products have a CAR containing the 4-1BB domain [2]. This is not coincidental, as 4-1BB is a key marker of T-cell activation. Its interaction with the 4-1BBL ligand and recruitment of various TRAF proteins initiates the p38 MAPK, AKT, and ERK signaling pathways. Consequently, transcription is activated from NF-κB-dependent promoters, increasing survivin, Bcl-XL, Bfl-1, and Bcl-2 production while reducing Bim levels [28, 29]. Moreover, 4-1BB signaling elevates the mitochondrial count and transmembrane potential, consequently improving aerobic processes in T-cells and augmenting their effector functions [30]. The 4-1BB TRAF-binding motifs, QEED and EEEE (*Fig. 3*), are involved in the interaction with TRAF1, TRAF2, TRAF3, and TRAF5 [31].

Incorporating 4-1BB into the CAR design improves the CAR T-cell ability to stay active, resulting in a phenotype closely aligned with central memory T-cells (Tcm). They exhibit low surface expression of PD-1, which is one of the most characteristic markers of T-cell exhaustion [32, 33]. This phenomenon can be partly accounted for by the metabolic shift towards enhanced mitochondrial processes and increased mitochondrial biogenesis induced by 4-1BB costimulation. Additionally, cells with CARs containing 4-1BB show elevated antiapoptotic gene expression and diminished pro-apoptotic factors. However, the activation of CAR T-cells is less pronounced with 4-1BB costimulation relative to CD28 [34]. This is explained by the recruitment of the THEMIS-SHP1 phosphatase tandem, which forms a complex with 4-1BB via a 10-AA motif at its C-terminus. Consequently, the resulting complex inhibits the phosphorylation of the CAR signaling domain: CD3ζ. Mutations in the QEED and EEEE motifs reduce cytokine secretion, the proportion of Tcm cells, and the antitumor activity of CAR T-cells [25, 35]. However, the incorporation of 4-1BB into CARs has been reported to lead to increased aggregation of CAR T-cells, which reduces their viability [36]. It is worth mentioning that deletion of the above-mentioned 10 AAs from the C-terminus of 4-1BB in this case prevents aggregation and restores the function of CAR T-cells. Furthermore, 4-1BB has been observed to elicit tonic signaling, resulting in CAR T-cell apoptosis [37]. Reducing the expression level of such CARs can enable the CAR T-cell activity to return to normal.

*OX40. *The OX40, a costimulatory receptor, is expressed on the surface of naïve T-cells (Tn) following their activation. The binding of OX40 to its ligand OX40L promotes the recruitment of TRAF2, 3, and 5 through the PIQEE motif (*Fig. 3*) [38, 39]. TRAF2, 3, and 5 adaptors induce the NF-κB signaling pathway, which promotes the synthesis of the antiapoptotic factors Bcl-XL and Bfl-1 in cells [40]. Also activated are the PI3K/AKT kinases involved in the synthesis of survivin and Aurora B kinase, inhibiting apoptosis and promoting T-cell proliferation [41, 42].

OX40 costimulation enhances the durability of second- generation CAR T-cells when compared to cells where CD28 and 4-1BB mediate costimulation in CAR constructs. However, the *in vivo *antitumor activity of CAR T-cells is largely unaffected by the CAR costimulatory domain. CAR T-cells with OX40-mediated costimulation exhibit enhanced target cell elimination* in vitro *[43]. Transcriptomic analysis of such CAR T-cells revealed upregulated expression of the genes responsible for DNA repair, oxidative phosphorylation, apoptosis inhibition, and memory differentiation and proliferation. According to existing data, the “specialization” of OX40 and 4-1BB implies that 4-1BB mainly enhances the development of CD8^+^ memory T-cells (Tm), while OX40 is biased towards CD4^+^ Tm cells [39]. Given that ICOS supports the differentiation of CD4^+^ T-cells into Th1, Th2, and Th17 effectors, the most effective costimulatory strategy for CD4^+^ CAR T-cells is likely to involve both ICOS and OX40. At the same time, the combination of CD28 and 4-1BB might be more suitable for the costimulation of CD8^+^ CAR T-cells.

*CD27. *CD27 is known to interact with the CD70 ligand, thereby facilitating T-cell proliferation and differentiation through the activation of the NF-κB, PI3K/AKT, and SAPK/JNK signaling pathways [44, 45]. Given that ICOS supports the differentiation of CD4^+^ T-cells into Th1, Th2, and Th17 effectors, the most effective costimulatory strategy for CD4^+^ CAR T-cells likely involves both ICOS and OX40. Thus, CD27 promotes the proliferation and viability of effector T-cells, as well as the generation of a Tm cell pool throughout the primary activation of Tn cells, during clonal expansion and at the effector stage (for example, in tumors). CD27 uses the functional motif PIQED(YR) and, possibly, EEEG (*Fig. 3*) to interact with TRAF2, TRAF3, and TRAF5 [45, 49]. A distinctive characteristic of CD27 compared to other TNFRSF family members is the formation of homodimers through disulfide bonds [49]. It is in this form that CD27 is present on the surface of resting T-cells, while their prolonged activation increases the proportion of the monomeric form, which probably protects T-cells from turning on costimulators during spontaneous activation.

Studies on the costimulatory potential of CD27 have shown that CD27 CAR T-cells can eradicate tumors more effectively than first-generation CAR T-cells, similar to CAR T-cells with CD28 or the 4-1BB costimulatory domain. The duration of CAR T-cell persistence, when costimulated with CD27, was found to be equivalent to that observed with 4-1BB costimulation [50, 51]. However, a direct comparison of the ability of second-generation CAR T-cells with either 4-1BB or CD27 to eliminate solid tumors in mice revealed superior antitumor activity for CD27 CAR T-cells [52]. The most effective configuration was determined to be a combination of three costimulatory domains, CD28, 4-1BB, and CD27, within the CAR. The enhanced proliferation, increased resistance to CAR loss, and reduced exhaustion were observed when compared to costimulation with one or two domains [53, 54].

*HVEM. *The Herpes Virus Entry Mediator, or HVEM, was first discovered as a receptor for herpes simplex virus-1 [55]. HVEM, an atypical member of its superfamily, exhibits binding capabilities to TNFSF molecules, specifically TNFSF14 and lymphotoxin-α, and to the immunoglobulin-like molecules CD272 and CD160 [56]. HVEM costimulates T-cells via trans-interaction, while cis-interaction inhibits costimulation by forming an isolated complex of HVEM with CD272 or CD160 [57]. Once activated, HVEM interacts with TRAF1, 2, 3, and 5, which triggers signaling via the NF-κB, JNK/AP-1, and PI3K/AKT pathways, thus resulting in heightened synthesis of cytokines and Bcl-2 [58, 59]. Consequently, effector properties, proliferation, and the viability of T-cells are enhanced. It is hypothesized that TRAF molecules interact with HVEM via the VTTVAVEET motif (*Fig. 3*), which partially aligns with the conserved motif (P/A/S/T/T)X(E/Q)E [58].

It is relatively recently that the potential of HVEMdependent costimulation of CAR T-cells has been evaluated [60, 61]. HVEM has been shown to combine the receptor properties of the IgSF and TNFRSF superfamilies. For instance, while CD28 facilitates the preferential differentiation of modified cells into effector memory T-cells (Tem), and 4-1BB into Tcm cells, HVEM leads to the development of a balanced population with almost identical proportions of both Tcm and Tem cells. Moreover, costimulation through CD28 primarily activates glycolytic metabolism, while 4-1BB activates oxidative phosphorylation. In contrast, HVEM enhances both metabolic pathways, establishing the most effective functional state of CAR T-cells. HVEM costimulation involvement, in comparison to CD28 and 4-1BB, likewise contributes to the minimal depletion of CAR T-cells. The greatest efficacy in solid tumors was achieved in mice treated with CAR T-cells expressing HVEM [61]. Additionally, the concurrent generation of CAR and the HVEM ligand TNFSF14 was found to enhance CAR T-cell infiltration into tumors because of significant chemokine secretion [62].

*TNFRSF18. *TNFRSF18, also known as GITR (glucocorticoid- induced TNFR-related protein), is constitutively expressed at low levels on the membrane of quiescent T-cells. Upon activation, the level of GITR on the T-cell surface increases significantly. GITR levels are found to be higher in Treg cells than in conventional T-cells, even without stimulation [63]. The interaction of GITR with its ligand GITRL weakens the immunosuppressive actions of Treg cells, and in effector T-cells, it boosts proliferation, cytokine release, and it has an antiapoptotic impact [64, 65]. Intracellular signaling from GITR involves interaction with TRAF1, 2, 3, 5 through the STED and PEEE motifs (*Fig. 3*) [66]. Stimulation of T-cells with antibodies targeting CD3, CD28, and GITR has been demonstrated to trigger both parallel responses, facilitating signaling synergism during costimulation, and unique effects, such as enhanced IL-27 production following GITR stimulation [67]. Costimulation via GITR primarily involves the NF-κB and MAPK signaling pathways [63].

In terms of tumor-killing efficacy, CAR T-cells costimulated via GITR are comparable to those based on CD28 and 4-1BB [68, 69]. Moreover, increased GITRL production by CAR T-cells improves cytokine secretion, tumor infiltration, and antitumor effects [70].

Some studies have focused on adding new costimulatory receptor parts from TNFRSF members, like BAFF-R, CD30, and CD40, into CARs [35, 71, 72]. CD40-mediated costimulation has been demonstrated to elicit a more robust NF-κB pathway activation compared to 4-1BB costimulation, potentially promoting enhanced *in vivo *persistence of CD40 CAR T-cells.

**Other costimulatory domains**

The focus on studying signaling pathways in diverse immune cells, such as natural killer cells and macrophages, has raised interest in costimulatory molecules that are not part of the immunoglobulin or TNF receptor superfamilies. Promising signaling molecules include Dap10 [73] and dectin-1 [74]. Contemporary genetic and cellular engineering methods seek to streamline the creation of CAR libraries featuring diverse combinations of costimulatory receptors or their components [71, 75]. Through the integration of high-throughput sequencing, a more detailed evaluation of the effects of diverse costimulators can be achieved, expanding beyond the extensively studied members of the IgSF and TNFRSF families, including the selection of specific combinations of costimulators for CD4^+^ or CD8^+^ T-cell populations.

## THE CD3ζ SIGNALING DOMAIN AND ITS ANALOGS

In the initial stages of development, the CD3ζ intracellular domain was the sole signaling domain incorporated into the CAR structure [76, 77]. This was due to the concept of the receptor itself, which was based on the combination of B- and T-cell receptors to target antigen recognition and subsequent T-cell activation. Early research, for example, revealed that ICD CD3ζ is appropriate for T-cell activation, thus laying the groundwork for CAR development [78]. CD3ζ became firmly “entrenched” in the receptor structure and “migrated” from generation to generation, providing the primary activation signal for CAR T-cells [79]. The inclusion of CD3ζ in all the CAR T-cell drugs approved so far for clinical application (*Fig. 1*) underscores the significance of this domain for developers, and, until recently, the lack of alternative options [5].

**Other CD3 group proteins**

Over time, interest in this part of CAR has increased significantly. In 2018, Sadelain et al. demonstrated that for full functionality of the CAR, only one active immunoreceptor tyrosine-based activation motif (ITAM) out of the three in CD3ζ is sufficient [80]. Its location and amino acid composition are both significant factors. The 1XX variant, with 1 indicating the position of the active ITAM relative to the cell membrane and X indicating an inactive ITAM, demonstrated the highest functionality in tumor elimination, whereas XX3 provided moderate support for CAR T-cell persistence. These data highlighted the necessity of reconsidering the role of the seemingly indispensable CD3ζ.

**Fig. 4:**
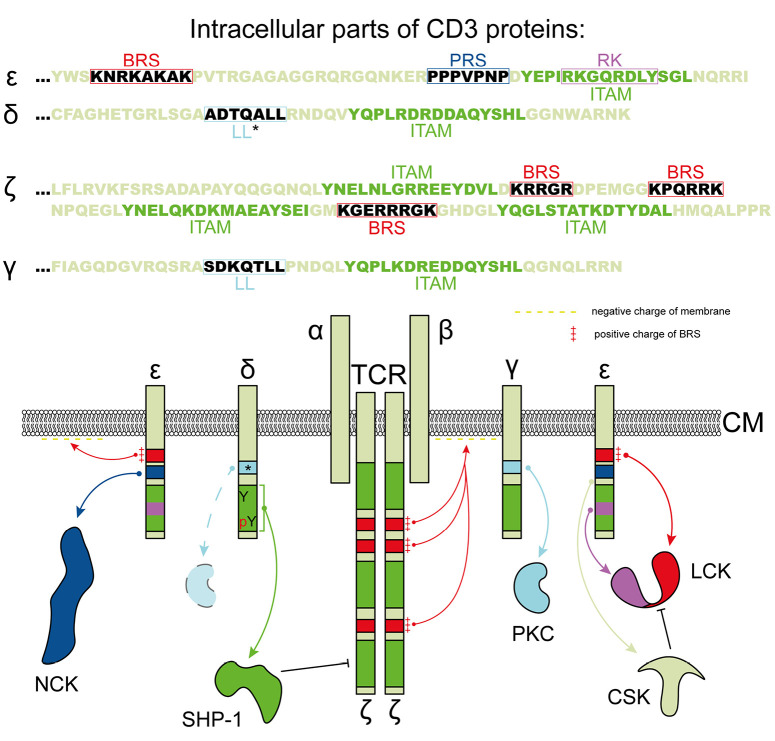
Structure and functional features of CD3 group proteins. Shown is the arrangement of intracellular CD3ε, δ, ζ, and γ domains, each possessing specific motifs for interacting with intracellular signaling partners. ζ, γ, δ, and ε – the representative members of the CD3 T-cell receptor family; ITAM – an immunoreceptor tyrosine-activating motif; BRS – a basic amino acid-rich site; PRS – a proline-rich site; RK – a receptor kinase motif; LL – a serine-dependent dileucine motif (* indicates the absence of serine upstream of LL, which reduces the involvement of LL in T-cell receptor regulation); TCR – a T-cell receptor; α, β – recognizing chains of the T-cell receptor; CM – cell membrane; Y – tyrosine; and pY – phosphorylated tyrosine

As a result, investigations were performed on possible analogs of CD3ζ; namely, other CD3 group representatives: ε, δ, and γ [81, 82]. In contrast to CD3ζ, the molecules within ICDs feature a single ITAM [83]. Although all ITAMs share the conserved YXXL/I-X6-8-YXXL/I sequence (X denotes any amino acid), the unique amino acid composition of each ITAM influences the binding affinity of signaling molecules (*Fig. 4*) [84].

In total, the TKR-CD3 multisubunit complex contains 10 ITAMs. Signal amplification likely results from a high concentration of tyrosine motifs, as a reduced number impairs TKR-CD3 complex function in mice [85]. Additionally, the variation between CD3 and the ITAMs within it is vital for signal transduction and the development of mature T-cells [86].

Beyond the unique amino acid sequences found in ITAM, the intracellular domains of each CD3 subunit exhibit distinctive characteristics (*Fig. 4*). The CD3ζ and CD3ε proteins feature segments rich in positively charged amino acids (basic-rich stretches), enabling their interaction with the inner membrane surface [87, 88]. The interaction of CD3ε with LCK kinase is mediated by ionic bonds between the BRS and acidic residues in the unique domain of LCK, and also through a receptor kinase motif (RK) and the SH3 domain of LCK [89, 90]. CD3ε also possesses a prolinerich sequence (PRS) that interacts with the adaptor protein NCK and is crucial for IC maturation and T-cell activation [91]. A proximal serine-dependent dileucine (SDKQTLL) motif within CD3γ participates in the decreasing of the number of TCRs on the cell membrane via a protein kinase C (PKC)-dependent mechanism [92]. In addition to ITAM, CD3δ contains a similar motif (ADTQALL), which lacks the serine needed for PKC interaction. Therefore, CD3δ is considered less significant in regulating the number of TKRs on the membrane than CD3γ [93].

Distinctive motifs within the structure of each representative CD3 protein are critical in the context of CARs, notwithstanding the fact that each CD3 variant alone may be sufficient for constructing a functional CAR structure. This fact has been illustrated by including CD3ε, δ, or γ into the CAR structure as a signaling domain instead of CD3ζ [81, 82].* In vivo*, CD3δ, CD3ε, or CD3γ CAR T-cells demonstrated superior tumor elimination efficacy compared to CD3ζ, this attributed to the distinct properties of specific CD3 group members. Thus, the binding of ICD CD3ε to CSK kinase suppresses LCK kinase activation, reducing the depletion of CAR T-cells and enabling their sustained presence. The binding of SHP-1 phosphatase to CD3δ monophosphorylated by a second tyrosine ICD results in a reduction in cytokine signaling and secretion intensity, presumably by mitigating the activation of the NF-κB pathway. The transcriptome analysis data demonstrated the Tm cell phenotype to be characterized by a reduction in glycolytic gene expression and an increase in mitochondrial metabolism gene expression. Additionally, TCF-1, known to be related to memory stem T-cells, is expressed at high levels in CD3δ [94]. Similar to Tn-cells, these cells can self-replicate most effectively and can transform into all kinds of memory cells [95]. Due to its greater proportion of positively charged CARs compared to acidic ones, CD3ε may bind more tightly to membrane phospholipids than CD3ζ does, which would decrease the availability of CARs to intracellular signaling partners, as has been noted with TKR and other proteins [88, 96]. As a result, the probability of both nonspecific and tonic signaling decreases. Incorporating a dimerizing CD8α hinge domain within the CAR structure revealed that dimeric CD3δ and CD3γ amplified cytokine release from CAR T-cells and elevated the surface expression of CD69 and 4-1BB, with the strongest effect observed upon mutation of the dileucine motifs (SDKQTAL and ADTQAAL) [81].

**TCR signaling partners**

A novel CAR format – designated bypassCARs (bCARs) – has been developed by investigating the distinct components of signaling cascades during TCR activation. Segments of the intracellular signaling molecules associated with T-cell receptors were incorporated into the bCAR structure, instead of ITAM-containing domains. The first bCAR-like chimeric molecules were developed at the end of the previous century to determine the key kinases essential for T-cell activation [97]. The structure of these receptors involved CD16. in combination with LCK, FYN, SYK, or ZAP70. Only within the SYK domain did modified cells exhibit the ability to lyse target cells in response to stimulation. Replacing CD16 with an scFv specific to a target antigen preserved the unique ability of SYK to activate the bCAR T-cell, bypassing the TCR [98].

Next, antitumor bCARs were constructed, which included CSK, FYN, the kinase domain of ZAP70 (ZAP70KD), LAT, SLP76, or PLCγ1, but lacked costimulatory domains [99]. While both ZAP70KDand PLCγ1-based bCARs activated modified T-cells, the PLCγ1 bCAR showed much weaker expression. ZAP70KD bCAR T-cells proved more efficient at removing solid tumors than CD3ζ CAR T-cells with a 4-1BB costimulatory domain *in vivo*. The ZAP70KDbased bCAR activated T-cells with knocked-out TCR and LCK, but not in the absence of SLP76 or LAT, confirming the preservation of the TCR downstream signaling pathway structure.

The recently developed second-generation bCARs incorporate an adapter domain from LAT or SLP76, which is comparable to the costimulatory domains found in traditional CARs. However, T-cells modified with such constructs exhibited excessively high levels of tonic signaling [100]. In particular, incorporating a CD28 signaling domain upstream of the ZAP70 kinase domain resulted in prolonged remission of B-cell tumors in mice treated with these bCAR T-cells, as opposed to conventional second-generation CAR T-cells with CD3ζ and a CD28 costimulatory domain.

## CONCLUSION

The investigation of CAR costimulatory and signaling domain combinations is a rapidly evolving field within CAR T-cell research, aiming to broaden the therapeutic application of these cells. The diversity across these domains opens a broad range of possibilities in the design of advanced CAR T-cells with enhanced functional attributes.

The analysis of the collected data suggests that selecting specific costimulatory domains has a significant impact on CAR T-cell activation, cytotoxicity, metabolic activity, in vivo persistence, and resistance to functional exhaustion. Consequently, the combination of various domains or the establishment of modular structures may potentially circumvent the critical limitations of existing methodologies in generating therapeutic CAR T-cell products and their applications, including tumor antigen heterogeneity, the immunosuppressive microenvironment, and the toxicity associated with adoptive transfer.

Furthermore, it is imperative to reduce the size of the CAR while maintaining its functionality and to identify a structure of minimal receptor activity, which should enhance the success of the modification and support increased and stable CAR receptor production by T-cells. Similar investigations are concurrently in progress [101].

Further optimization of CAR T-cell signaling domains requires a deeper understanding of T-lymphocyte activation mechanisms, along with the use of advanced technologies such as CRISPR screening, transcriptomics, proteomics, and computational modeling [75, 102, 103, 104, 105]. This will enable the creation of personalized cellular products precisely tailored to the biology of a specific tumor type. Alongside improvements in receptor generation and the development of modular systems [106, 107, 108], research in this area could lead to groundbreaking therapeutic solutions, expanding the applications of CAR T-cell technology and enhancing its effectiveness in treating both cancer and, potentially, autoimmune and infectious diseases.

## Abbreviations

CAR: chimeric antigen receptor
CAR T-cells: chimeric antigen receptor-modified T-cells
IS: immune synapse
TAA: tumor-associated antigen
FASL: Fas ligand
FAS: Fas receptor
ICD: intracellular domain
scFv: single-chain variable fragment
VHH: variable domain of heavy-chain antibody
CD: cluster of differentiation
BCMA: B-cell maturation antigen
IgSF: immunoglobulin superfamily
TNFRSF: tumor necrosis factor receptor superfamily
TNFSF: tumor necrosis factor superfamily
APC: antigen-presenting cell
MHC I/II: major histocompatibility complex class I and II
TCR: T-cell receptor
α, β: T-cell receptor recognition chains
ζ, γ, δ, ε: CD3 proteins of the T-cell receptor
ITAM: immunoreceptor tyrosine-based activation motif
AA: amino acid
Y: tyrosine
Tn cells: naïve T-cells
ICOSL: inducible T-cell costimulator ligand
IL: interleukin
IFN-γ: interferon gamma
TNF-α: tumor necrosis factor alpha
Treg cells: regulatory T-cells
FAP: fibroblast activation protein
Th cells: helper T-cells
Tm cells: memory T-cells
TRAF: TNF receptor-associated factor
Tcm cells: central memory T-cells
HVEM: herpesvirus entry mediator
Tem cells: effector memory T-cells
GITR: glucocorticoid-induced TNF receptor-related protein
BRS: basic residue-rich sequences (in some CD3 molecules)
RK: receptor kinase
PRS: proline-rich sequence of CD3ε
PKC: protein kinase C
bCAR: chimeric antigen receptors with signaling domains represented by parts of various intracellular signaling partners of the TCR
ZAP70KD: kinase domain of ZAP70

## References

[1] Yazbeck V., Alesi E., Myers J. (2022). An overview of chemotoxicity and radiation toxicity in cancer therapy. Advances in Cancer Research.. Elsevier;.

[2] Cappell KM., Kochenderfer JN. (2023). Long-term outcomes following CAR T cell therapy: what we know so far.. Nat Rev Clin Oncol..

[3] Li Q., Lei X., Zhu J. (2023). Radiotherapy/chemotherapy-immunotherapy for cancer management: From mechanisms to clinical implications.. Oxid Med Cell Longev..

[4] McLellan AD., Ali Hosseini Rad SM. (2019). Chimeric antigen receptor T cell persistence and memory cell formation.. Immunol Cell Biol..

[5] Mitra A., Barua A., Huang L. (2023). From bench to bedside: the history and progress of CAR T cell therapy.. Front Immunol..

[6] Finney HM., Lawson ADG., Bebbington CR., Weir ANC. (1998). Chimeric receptors providing both primary and costimulatory signaling in T cells from a single gene product.. J Immunol..

[7] Krause A., Guo HF., Latouche JB. (1889). Antigen-dependent CD28 signaling selectively enhances survival and proliferation in genetically modified activated human primary T lymphocytes.. J Exp Med..

[8] Esensten JH., Helou YA., Chopra G. (2016). CD28 costimulation: From mechanism to therapy.. Immunity..

[9] Honikel MM., Olejniczak SH. (2022). Co-stimulatory receptor signaling in CAR-T cells.. Biomolecules..

[10] Kunkl M., Sambucci M., Ruggieri S. (2019). CD28 autonomous signaling up-regulates C-myc expression and promotes glycolysis enabling inflammatory T cell responses in multiple sclerosis.. Cells..

[11] Kawalekar OU., O’Connor RS., Fraietta JA. (2016). Distinct signaling of coreceptors regulates specific metabolism pathways and impacts memory development in CAR T cells.. Immunity..

[12] Guedan S., Madar A., Casado-Medrano V. (2020). Single residue in CD28-costimulated CAR-T cells limits long-term persistence and antitumor durability.. J Clin Invest..

[13] Boucher JC., Li G., Kotani H. (2021). CD28 costimulatory domain-targeted mutations enhance chimeric antigen receptor T-cell function.. Cancer Immunol Res..

[14] Kofler DM., Chmielewski M., Rappl G. (2011). CD28 costimulation Impairs the efficacy of a redirected t-cell antitumor attack in the presence of regulatory t cells which can be overcome by preventing Lck activation.. Mol Ther..

[15] Gulati P., Rühl J., Kannan A. (2018). Aberrant lck signal via CD28 costimulation augments antigen-specific functionality and tumor control by redirected T cells with PD-1 blockade in humanized mice.. Clin Cancer Res..

[16] Ferreira LMR., Muller YD. (2021). CAR T-cell therapy: Is CD28-CAR heterodimerization its Achilles’ heel?. Front Immunol..

[17] Yoshinaga SK., Whoriskey JS., Khare SD. (1999). T-cell co-stimulation through B7RP-1 and ICOS.. Nature.

[18] van Berkel MEAT., Oosterwegel MA. (2006). CD28 and ICOS: similar or separate costimulators of T cells. Immunol Lett..

[19] Paulos CM., Carpenito C., Plesa G. (2010). The inducible costimulator (ICOS) is critical for the development of human TH17 cells.. Sci Transl Med..

[20] Parry RV., Rumbley CA., Vandenberghe LH. (2003). CD28 and inducible costimulatory protein Src homology 2 binding domains show distinct regulation of phosphatidylinositol 3-kinase, Bcl-xL, and IL-2 expression in primary human CD4 T lymphocytes.. J Immunol..

[21] Fos C., Salles A., Lang V. (2008). ICOS ligation recruits the p50alpha PI3K regulatory subunit to the immunological synapse.. J Immunol..

[22] Shen C-J., Yang Y-X., Han EQ. (2013). Chimeric antigen receptor containing ICOS signaling domain mediates specific and efficient antitumor effect of T cells against EGFRvIII expressing glioma.. J Hematol Oncol..

[23] Guedan S., Chen X., Madar A. (2014). ICOS-based chimeric antigen receptors program bipolar TH17/TH1 cells.. Blood..

[24] Wyatt MM., Huff LW., Nelson MH. (2023). Augmenting TCR signal strength and ICOS costimulation results in metabolically fit and therapeutically potent human CAR Th17 cells.. Mol Ther..

[25] Fujiwara K., Kitaura M., Tsunei A. (2021). Structure of the signal transduction domain in second-generation CAR regulates the input efficiency of CAR signals.. Int J Mol Sci..

[26] Vanamee ÉS., Faustman DL. (2018). Structural principles of tumor necrosis factor superfamily signaling.. Sci Signal..

[27] Ye H., Park YC., Kreishman M. (1999). The structural basis for the recognition of diverse receptor sequences by TRAF2.. Mol Cell..

[28] Ward-Kavanagh LK., Lin WW., Šedý JR., Ware CF. (2016). The TNF receptor superfamily in co-stimulating and co-inhibitory responses.. Immunity..

[29] Craxton A., Draves KE., Gruppi A., Clark EA. (2005). BAFF regulates B cell survival by downregulating the BH3-only family member Bim via the ERK pathway.. J Exp Med..

[30] Teijeira A., Labiano S., Garasa S. (2018). Mitochondrial morphological and functional reprogramming following CD137 (4-1BB) costimulation.. Cancer Immunol Res..

[31] Glez-Vaz J., Azpilikueta A., Ochoa MC. (2023). CD137 (4-1BB) requires physically associated cIAPs for signal transduction and antitumor effects.. Sci Adv..

[32] Cappell KM., Kochenderfer JN. (2021). A comparison of chimeric antigen receptors containing CD28 versus 4-1BB costimulatory domains.. Nat Rev Clin Oncol..

[33] Boroughs AC., Larson RC., Marjanovic ND. (2020). A distinct transcriptional program in human CAR T cells bearing the 4-1BB signaling domain revealed by scRNA-seq.. Mol Ther..

[34] Sun C., Shou P., Du H. (2020). THEMIS-SHP1 recruitment by 4-1BB tunes LCK-mediated priming of chimeric antigen receptor-redirected T cells.. Cancer Cell..

[35] Mamonkin M., Mukherjee M., Srinivasan M. (2018). Reversible transgene expression reduces fratricide and permits 4-1BB costimulation of CAR T cells directed to T-cell malignancies.. Cancer Immunol Res..

[36] Dou Z., Bonacci TR., Shou P. (2024). 4-1BB-encoding CAR causes cell death via sequestration of the ubiquitin-modifying enzyme A20.. Cell Mol Immunol..

[37] Gomes-Silva D., Mukherjee M., Srinivasan M. (2017). Tonic 4-1BB costimulation in chimeric antigen receptors impedes T cell survival and is vector-dependent.. Cell Rep..

[38] Willoughby J., Griffiths J., Tews I., Cragg MS. (2017). OX40: Structure and function - What questions remain.. Mol Immunol. 2017;83:13–22..

[39] Croft M. (2003). Costimulation of T cells by OX40, 4-1BB, and CD27.. Cytokine Growth Factor Rev..

[40] Kawamata S., Hori T., Imura A. (1998). Activation of OX40 signal transduction pathways leads to tumor necrosis factor receptor-associated factor (TRAF) 2- and TRAF5-mediated NF-κB activation.. J Biol Chem..

[41] Croft M. (2010). Control of immunity by the TNFR-related molecule OX40 (CD134).. Annu Rev Immunol..

[42] Song J., So T., Croft M. (2008). Activation of NF-kappaB1 by OX40 contributes to antigen-driven T cell expansion and survival.. J Immunol..

[43] Tan J., Jia Y., Zhou M. (2022). Chimeric antigen receptors containing the OX40 signalling domain enhance the persistence of T cells even under repeated stimulation with multiple myeloma target cells.. J Hematol Oncol..

[44] Starzer AM., Berghoff AS. (2020). New emerging targets in cancer immunotherapy: CD27 (TNFRSF7).. ESMO Open..

[45] Akiba H., Nakano H., Nishinaka S. (1998). CD27, a member of the tumor necrosis factor receptor superfamily, activates NF-kappaB and stress-activated protein kinase/c-Jun N-terminal kinase via TRAF2, TRAF5, and NF-kappaB-inducing kinase.. J Biol Chem..

[46] Dolfi DV., Boesteanu AC., Petrovas C. (2008). Late signals from CD27 prevent Fas-dependent apoptosis of primary CD8+ T cells.. J Immunol..

[47] van de Ven K., Borst J. (2015). Targeting the T-cell co-stimulatory CD27/CD70 pathway in cancer immunotherapy: rationale and potential.. Immunotherapy..

[48] Peperzak V., Veraar EAM., Keller AM. (2010). The Pim kinase pathway contributes to survival signaling in primed CD8+ T cells upon CD27 costimulation.. J Immunol..

[49] Yamamoto H., Kishimoto T., Minamoto S. (1998). NF-κB activation in CD27 signaling: Involvement of TNF receptor-associated factors in its signaling and identification of functional region of CD27.. J Immunol..

[50] Song D-G., Ye Q., Poussin M. (2012). CD27 costimulation augments the survival and antitumor activity of redirected human T cells in vivo.. Blood..

[51] Song D-G., Powell DJ. (2012). Pro-survival signaling via CD27 costimulation drives effective CAR T-cell therapy.. Oncoimmunology..

[52] Han Y., Xie W., Song D-G., Powell DJ., Jr. I.O. (2018). Control of triple-negative breast cancer using ex vivo self-enriched, costimulated NKG2D CAR T cells.. J Hematol Oncol..

[53] Zhang C., Jia J., Heng G. (2023). CD27 agonism coordinates with CD28 and 4-1BB signal to augment the efficacy of CAR-T cells in colorectal tumor.. Med Oncol..

[54] Supimon K., Sangsuwannukul T., Sujjitjoon J. (2021). Anti-mucin 1 chimeric antigen receptor T cells for adoptive T cell therapy of cholangiocarcinoma.. Sci Rep..

[55] Montgomery RI., Warner MS., Lum BJ., Spear PG. (1996). Herpes simplex virus-1 entry into cells mediated by a novel member of the TNF/NGF receptor family.. Cell..

[56] Šedý JR., Ramezani-Rad P. (2019). HVEM network signaling in cancer.. Advances in Cancer Research..

[57] Steinberg MW., Cheung TC., Ware CF. (2011). The signaling networks of the herpesvirus entry mediator (TNFRSF14) in immune regulation.. Immunol Rev..

[58] Hsu H., Solovyev I., Colombero A. (1997). ATAR, a novel tumor necrosis factor receptor family member, signals through TRAF2 and TRAF5.. J Biol Chem..

[59] Soroosh P., Doherty TA., So T. (2011). Herpesvirus entry mediator (TNFRSF14) regulates the persistence of T helper memory cell populations.. J Exp Med..

[60] Nunoya J-I., Masuda M., Ye C., Su L. (2019). Chimeric antigen receptor T cell bearing herpes virus entry mediator co-stimulatory signal domain exhibits high functional potency.. Mol Ther Oncolytics..

[61] Sun S., Huang C., Lu M. (2023). Herpes virus entry mediator costimulation signaling enhances CAR T-cell efficacy against solid tumors through metabolic reprogramming.. Cancer Immunol Res..

[62] Zhang N., Liu X., Qin J. (2023). LIGHT/TNFSF14 promotes CAR-T cell trafficking and cytotoxicity through reversing immunosuppressive tumor microenvironment.. Mol Ther..

[63] Azuma M. (2019). Co-signal molecules in T-cell activation: Historical Overview and Perspective.. Adv Exp Med Biol..

[64] Tian J., Zhang B., Rui K., Wang S. (2020). The role of GITR/GITRL interaction in autoimmune diseases.. Front Immunol..

[65] Ronchetti S., Zollo O., Bruscoli S. (2004). GITR, a member of the TNF receptor superfamily, is costimulatory to mouse T lymphocyte subpopulations.. Eur J Immunol..

[66] So T., Nagashima H., Ishii N. (2015). TNF receptor-associated factor (TRAF) signaling network in CD4(+) T-lymphocytes.. Tohoku J Exp Med..

[67] Kanamaru F., Youngnak P., Hashiguchi M. (2004). Costimulation via glucocorticoid-induced TNF receptor in both conventional and CD25+ regulatory CD4+ T cells.. J Immunol..

[68] Xi B., Berahovich R., Zhou H. (2019). A real-time potency assay for chimeric antigen receptor T cells targeting solid and hematological cancer cells.. J Vis Exp..

[69] Golubovskaya VM. (2018). GITR domain inside CAR co-stimulates activity of CAR-T cells against cancer.. Front Biosci..

[70] Wang Y., Wang L., Seo N. (2023). CAR-modified Vγ9Vδ2 T cells propagated using a novel bisphosphonate prodrug for allogeneic adoptive immunotherapy.. Int J Mol Sci..

[71] Goodman DB., Azimi CS., Kearns K. (2022). Pooled screening of CAR T cells identifies diverse immune signaling domains for next-generation immunotherapies.. Sci Transl Med..

[72] Levin-Piaeda O., Levin N., Pozner S. (2021). The intracellular domain of CD40 is a potent costimulatory element in chimeric antigen receptors.. J Immunother..

[73] Li S., Zhao R., Zheng D. (2022). DAP10 integration in CAR-T cells enhances the killing of heterogeneous tumors by harnessing endogenous NKG2D.. Mol Ther Oncolytics..

[74] Liang X., Huang Y., Li D. (2023). Distinct functions of CAR-T cells possessing a dectin-1 intracellular signaling domain.. Gene Ther..

[75] Daniels KG., Wang S., Simic MS. (2022). Decoding CAR T cell phenotype using combinatorial signaling motif libraries and machine learning.. Science..

[76] Gross G., Waks T., Eshhar Z. (1989). Expression of immunoglobulin-T-cell receptor chimeric molecules as functional receptors with antibody-type specificity.. Proc Natl Acad Sci U S A..

[77] Kuwana Y., Asakura Y., Utsunomiya N. (1987). Expression of chimeric receptor composed of immunoglobulin-derived V resions and T-cell receptor-derived C regions.. Biochem Biophys Res Commun..

[78] Eshhar Z., Waks T., Gross G., Schindler DG. (1993). Specific activation and targeting of cytotoxic lymphocytes through chimeric single chains consisting of antibody-binding domains and the gamma or zeta subunits of the immunoglobulin and T-cell receptors.. Proc Natl Acad Sci U S A..

[79] Zheng Z., Li S., Liu M. (2023). Fine-tuning through generations: Advances in structure and production of CAR-T therapy.. Cancers (Basel)..

[80] Feucht J., Sun J., Eyquem J. (2018). Calibrated CAR activation potential directs alternative T cell fates and therapeutic potency.. Blood..

[81] Velasco Cárdenas RMH., Brandl SM., Meléndez AV. (2023). Harnessing CD3 diversity to optimize CAR T cells.. Nat Immunol..

[82] Wang P., Wang Y., Zhao X. (2025). Chimeric antigen receptor with novel intracellular modules improves antitumor performance of T cells.. Signal Transduct Target Ther..

[83] Reth M. (1989). Antigen receptor tail clue.. Nature.

[84] Love PE., Hayes SM. (2010). ITAM-mediated signaling by the T-cell antigen receptor.. Cold Spring Harb Perspect Biol..

[85] Holst J., Wang H., Eder KD. (2008). Scalable signaling mediated by T cell antigen receptor-CD3 ITAMs ensures effective negative selection and prevents autoimmunity.. Nat Immunol..

[86] Bettini ML., Chou P-C., Guy CS. (2017). Cutting edge: CD3 ITAM diversity is required for optimal TCR signaling and thymocyte development.. J Immunol..

[87] Aivazian D., Stern LJ. (2000). Phosphorylation of T cell receptor zeta is regulated by a lipid dependent folding transition.. Nat Struct Biol..

[88] Xu C., Gagnon E., Call ME. (2008). Regulation of T cell receptor activation by dynamic membrane binding of the CD3ε cytoplasmic tyrosine-based motif.. Cell..

[89] Li L., Guo X., Shi X. (2017). Ionic CD3−Lck interaction regulates the initiation of T-cell receptor signaling.. Proc Natl Acad Sci U S A..

[90] Hartl FA., Beck-Garcìa E., Woessner NM. (2020). Noncanonical binding of Lck to CD3ε promotes TCR signaling and CAR function.. Nat Immunol..

[91] Gil D., Schamel WWA., Montoya Ma. (2002). Recruitment of nck by CD3ε reveals a ligand-induced conformational change essential for T cell receptor signaling and synapse formation..

[92] Dietrich J., Hou X., Wegener AM., Geisler C. (1994). CD3 gamma contains a phosphoserine-dependent di-leucine motif involved in down-regulation of the T cell receptor.. EMBO J..

[93] Wegener A-MK., Hou X., Dietrich J., Geisler C. (1995). Distinct domains of the CD3-γ chain are involved in surface expression and function of the T cell antigen receptor.. J Biol Chem..

[94] Escobar G., Mangani D., Anderson AC. (2020). T cell factor 1: A master regulator of the T cell response in disease.. Sci Immunol..

[95] Gattinoni L., Speiser DE., Lichterfeld M., Bonini C. (2017). T memory stem cells in health and disease.. Nat Med..

[96] Yeung T., Gilbert GE., Shi J. (2008). Membrane phosphatidylserine regulates surface charge and protein localization.. Science..

[97] Kolanus W., Romeo C., Seed B. (1993). T cell activation by clustered tyrosine kinases.. Cell..

[98] Fitzer-Attas CJ., Schindler DG., Waks T., Eshhar Z. (1998). Harnessing Syk family tyrosine kinases as signaling domains for chimeric single chain of the variable domain receptors: optimal design for T cell activation.. J Immunol..

[99] Tousley AM., Rotiroti MC., Labanieh L. (2023). Co-opting signalling molecules enables logic-gated control of CAR T cells.. Nature.

[100] Balagopalan L., Moreno T., Qin H. (2024). Generation of antitumor chimeric antigen receptors incorporating T cell signaling motifs.. Sci Signal..

[101] Si W., Fan Y-Y., Qiu S-Z. (2023). Design of diversified chimeric antigen receptors through rational module recombination.. iScience..

[102] Salter AI., Ivey RG., Kennedy JJ. (2018). Phosphoproteomic analysis of chimeric antigen receptor signaling reveals kinetic and quantitative differences that affect cell function.. Sci Signal..

[103] Ramello MC., Benzaïd I., Kuenzi BM. (2019). An immunoproteomic approach to characterize the CAR interactome and signalosome.. Sci Signal..

[104] Qiu S., Chen J., Wu T. (2024). CAR-Toner: an AI-driven approach for CAR tonic signaling prediction and optimization.. Cell Res..

[105] Rohrs JA., Zheng D., Graham NA. (2018). Computational model of chimeric antigen receptors explains site-specific phosphorylation kinetics.. Biophys J..

[106] Sheykhhasan M., Ahmadieh-Yazdi A., Vicidomini R. (2024). CAR T therapies in multiple myeloma: unleashing the future.. Cancer Gene Ther..

[107] Stepanov AV., Xie J., Zhu Q. (2024). Control of the antitumour activity and specificity of CAR T cells via organic adapters covalently tethering the CAR to tumour cells.. Nat Biomed Eng..

[108] Stepanov AV., Kalinin RS., Shipunova VO. (2022). Switchable targeting of solid tumors by BsCAR T cells.. Proc Natl Acad Sci U S A..

